# Supplementary material for: Global land and water limits to electrolytic hydrogen production using wind and solar resources
Source: Nat Commun. 2023 Sep 8;14:5532. doi: 10.1038/s41467-023-41107-x (PMC10491841; doi:10.1038/s41467-023-41107-x)
Supplement: Supplementary file 1 — Supplementary Information [file 41467_2023_41107_MOESM1_ESM.pdf]

SUPPLEMENTARY INFORMATION

S.1 Hydrogen demand and production

S.1.1 Hydrogen demand

The electrolytic hydrogen demand analyzed in this study is independent of the power production sector, due to the difficulty of predicting to which extent countries will adopt hydrogen for seasonal storage and as fuel in power production technologies. While there is currently no use of hydrogen in the power sector as of 2020, it is projected that by 2050, the power sector could contribute to a global increase in hydrogen demand of 102 Mt/y [1], representing a 24% growth compared to the demand estimated in this study). However, several factors, such as the availability of salt caverns for geological seasonal storage [2] and competition with other storage technologies, will influence the deployment of hydrogen as a storage solution. Hydrogen can also be utilized as a fuel in gas turbines for power production [3], but its different combustion properties necessitate the replacement or retrofitting of existing gas turbines. While it is feasible to quantify the hydrogen demand per country based on the gas turbines in operation in 2020 [4], there is no assurance that these technologies will maintain the same share of power production in each country till 2050. **Figure S.1** provides an overview of the steps involved in determining the country- and sector-specific hydrogen demand considered in this study. **Table S.1** presents the global production of ammonia and methanol, and their associated hydrogen demand calculated following the steps outlined in **Figure S.1 (a)** (**Methods - Section 5.1 - Hydrogen demand**).

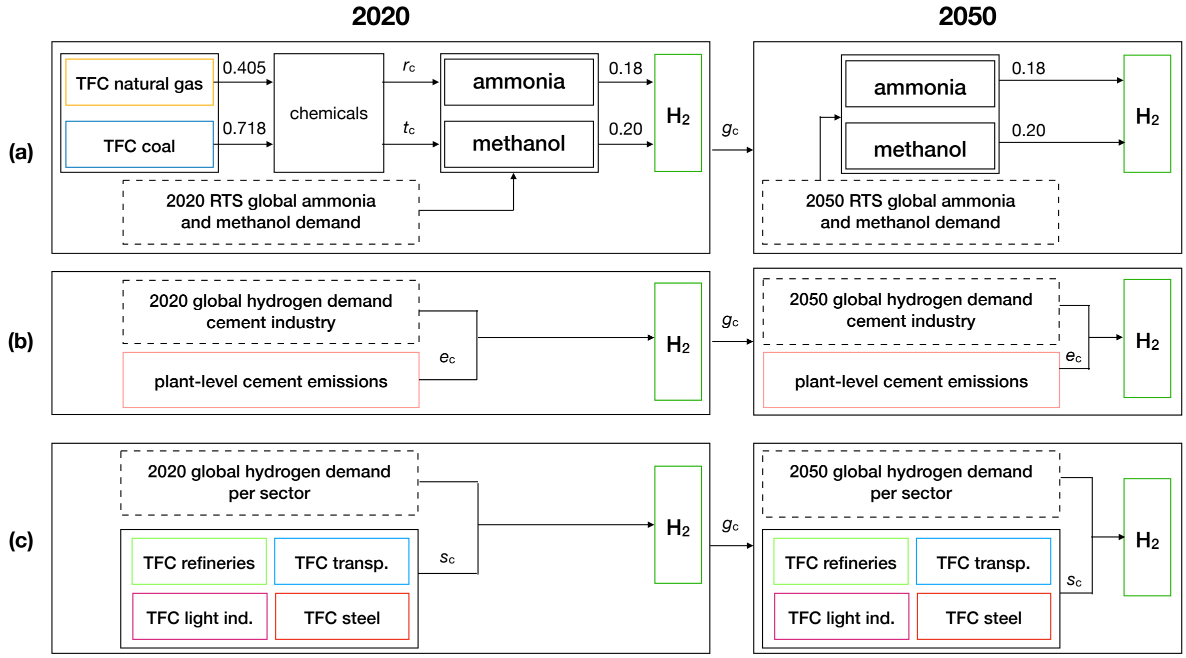

Figure S.1: Steps for the computation of the hydrogen demand in 2020 and 2050 per country. This figure outlines the step-by-step process used to compute the hydrogen demand for each country in 2020 and 2050. The calculations rely on the total final energy consumption (TFC) data sourced from the historical World Energy Balances provided by the International Energy Agency (IEA) [5], as well as emissions data from cement plants [6,7,8]. The parameters r_c and t_c represent the fraction of ammonia and methanol of the total production of chemicals in a given country c, e_c is the fraction of emissions from the cement industry per

country and s_c signifies the fraction of TFC per sector and per country, including refineries, transportation, steel and light industry. Additionally, g_c reflects the population variation relative to country c between 2020 and 2050.

Table S.1: Global production of ammonia and methanol in 2020 and 2050 with corresponding hydrogen demand.

feedstocks (Mt/y)	ammonia [9]	hydrogen for ammonia	methanol [9]	hydrogen for methanol
**2020**	189	34	100	20
**2050**	243	44	181	36

S.1.2 Alternative low-carbon hydrogen production methods

Despite the availability of various production methods, hydrogen generated through water electrolysis from wind and solar power is associated with the lowest process CO_2 emissions [10]. However, a country's potential as a large-scale, low-carbon hydrogen producer relies on several crucial factors, including its renewable power potential, the amount of available land, and water availability [11]. Other technologies for low-carbon hydrogen production, such as steam methane reforming coupled with carbon capture (or blue hydrogen) and pyrolysis (or turquoise hydrogen) are also considered. The carbon intensity of these methods depends on the extent of methane system leakage, which currently varies widely among and within countries. The leakage rate ranges from less than 0.5% in countries like Norway, the UK, and the Netherlands, to over 2% in regions within the United States, Russia, Algeria or Libya [12]. Furthermore, measurements indicate that emissions from Permian basins alone account for more than 9% of gross gas production [13].

S.1.3 Country-specific hydrogen strategies

In the following section, we examine the hydrogen strategies of four prominent countries that are projected to experience substantial increases in hydrogen demand between 2020 and 2050.

China [14]

China primarily relies on coal gasification for hydrogen production, in contrast to other countries where natural gas is the main source. China's initial hydrogen roadmap emphasized fuel cell vehicles and included purchase subsidies for light-, medium-, and heavy-duty vehicles. The country aimed to deploy over 33 000 fuel cell vehicles across five clusters of cities in a four-year demonstration project, which later increased to 50 000 vehicles by 2025. Recent plans involve producing 0.1 - 0.2 Mt/y of electrolytic hydrogen by 2025. The China Hydrogen Alliance predicts total hydrogen use to reach 130 Mt/y by 2060, accounting for 20% of China's total final energy consumption.

Australia [15,16]

Australia aims to become one of the top three hydrogen-exporting countries to Asian markets by 2030. As part of its net-zero goals, Australia plans to produce clean hydrogen at a cost below 2 $/kg. The country has allocated 464 M$ to establish clean hydrogen industrial hubs. The Australian national hydrogen strategy

includes measures to support research and development of hydrogen supply chains and create favorable regulatory conditions.

France and other European countries [17,18,19,20,21,22]

The French hydrogen strategy is aligned with the other three major economies in the European Economic Area. France, Italy, Spain, and Germany plan the installation of 6.5 GW, 5 GW, 4 GW, and 5 GW of low-carbon hydrogen production plants by 2030, respectively. At the European scale, the RePowerEU Plan from the European Commission set a production target of 10 Mt/y by 2030, in addition to 10 Mt/y of hydrogen import. Among them, Belgium plans to base 100% of its supply on import due to the limited local renewable energy potential.

Japan [23,24]

On the supply side, Japan will develop international commercial-scale supply chains by 2030 to import 0.3 Mt/y of hydrogen. On the demand side, the Green Growth Strategy established the core targets of 3 Mt/y of hydrogen consumption by 2030 and 20 Mt/y by 2050. Additional actions include commercializing the use of hydrogen in the power sector, in energy-intensive industries, and in heavy-duty vehicles. Furthermore, the installation of stationary fuel cells for the off-grid supply of electricity and the development of liquefied hydrogen carrier vessels are part of the actions. The Japanese Green Growth Strategy has two additional targets: introducing 30% of hydrogen blending in gas-fired power plants and 20% ammonia-blending in coal-fired power plants to cover 1% of Japan's power generation. As a net importer of hydrogen, Japan has developed an international supply chain with Brunei and Australia.

United States [25,26,27]

The national hydrogen strategy of the United States builds on the Infrastructure Investment and Jobs Act (November 2021), the Hydrogen Energy Earthshot (June 2021) and the Inflation Reduction Act (August 2022). Scenarios have been developed indicating 10 Mt of hydrogen demand by 2030, 20 Mt/y by 2040 and 50 Mt/y by 2050. The strategy builds around three targets: (i) clean hydrogen use in the highest-value applications, (ii) reduction of the cost of clean hydrogen production, and (iii) creation of clean hydrogen hubs for large-scale production. The strategy aims at lowering the cost of hydrogen production to 2 $/kg by 2025 and 1 $/kg by 2030. More recently, the Inflation Reduction Act (IRA) has created a wide array of energy tax incentives providing a production tax credit of up to 3 $/kg for low-carbon hydrogen covering the first 10 years of operation of a production plant.

S.2 Electricity demand

Electricity demand in 2050 is derived with a two-step approach (**Methods, Section 5.2**). Here we provide numerical values for sample countries, at the intermediate step (**step (i)**) and at two different final steps (**step (ii)**, **(ii')**):

- **step (i):** electricity demand in 2050 is predicted based only on the variation in population from the demand in 2020,
- **step (ii'):** electricity demand in 2050 is predicted from the results of an energy-system model at a regional level, then disaggregated at a country level based on the electricity demand in 2020 (ignoring **step (i)**),
- **step (ii):** electricity demand in 2050 is predicted from the results of an energy-system model at a regional level, then disaggregated at a country level based on the estimates of demand for 2050 from **step (i)**,

Table S.2: Estimation of electricity demand in sample countries. The computation of electricity demand in 2050 can be approached in various ways using historical data. In this table, we present numerical values that support the two-step approach adopted in this study.

**country -** *c*	**2020 electricity demand (TWh/y)**	**2050 electricity demand (TWh/y)**	**electricity demand variation relative to 2020**	**population variation relative to 2020**
Trinidad and Tobago	(i) 8.24<br>(ii) 8.24<br>(ii') 8.24	(i) 7.91<br>(ii') 11.81<br>(ii) 9.80	(i) -4%<br>(ii') +43%<br>(ii) +19%	-4%
Brunei	(i) 4.18<br>(ii) 4.18<br>(ii') 4.18	(i) 4.71<br>(ii') 9.17<br>(ii) 10.19	(i) +13%<br>(ii') +119%<br>(ii) +144%	+13%
Canada	(i) 557.51<br>(ii) 557.51<br>(ii') 557.51	(i) 674.61<br>(ii') 799.17<br>(ii) 835.51	(i) +21%<br>(ii') +43%<br>(ii) +50%	+21%

In the case of Trinidad and Tobago, if the prediction of electricity demand in 2050 were based on the demand in 2020 [28], adjusted for the change in population [29] between 2020 and 2050 (**step (i)**), the estimated electricity demand in 2050 would be 7.91 TWh/y. This would indicate a 4% decrease in electricity demand compared to 2020, in line with the expected population change (**step (i)**). In **step (ii')**, the prediction of electricity demand for 2050 relies on projections from an energy system model at regional scale [30]. The country-specific electricity demand is determined by disaggregating the regional values for 2050 [30] to the country level, proportionally to the demand in 2020 [31] within the countries in the same region. For Trinidad and Tobago, this implies a demand increase of 43% between 2020 and 2050. However, this introduces an error in the country level disaggregation, as it assumes that the country's weight in demand within the region remains the same in 2050 as in 2020. To address this issue, **step (ii)** combines **step (i)** and **step (ii')**. In the case of Trinidad and Tobago, the increase in electricity demand of +43% derived from the energy system model prediction at the regional scale is offset by the lower weight of the country within the region, resulting from the decline in population (-4%). As a result, the overall variation in electricity demand is +19%. Both Brunei and Canada expect a population increase of +13% and +21% respectively between 2020 and 2050 [29]. The variation in electricity demand based on the energy system model at the regional scale is +119% and +43% [30] for Brunei and Canada, respectively. Consequently, the combination of larger weight in the regional disaggregation (due to the demographic increase) and the increase in electricity demand at the regional scale leads to a projected electricity demand increase of +144% for Brunei and 50% for Canada.

S.3 Power production

Figure S.2 illustrates the global production potential of solar and wind technologies, calculated using a yearly-average geographical discretization at a grid resolution of 0.75° × 0.75° (approximately 80 km × 80 km at the Equator). Further information on the methodology can be found in **Methods - Section 5.3 Energy production**.

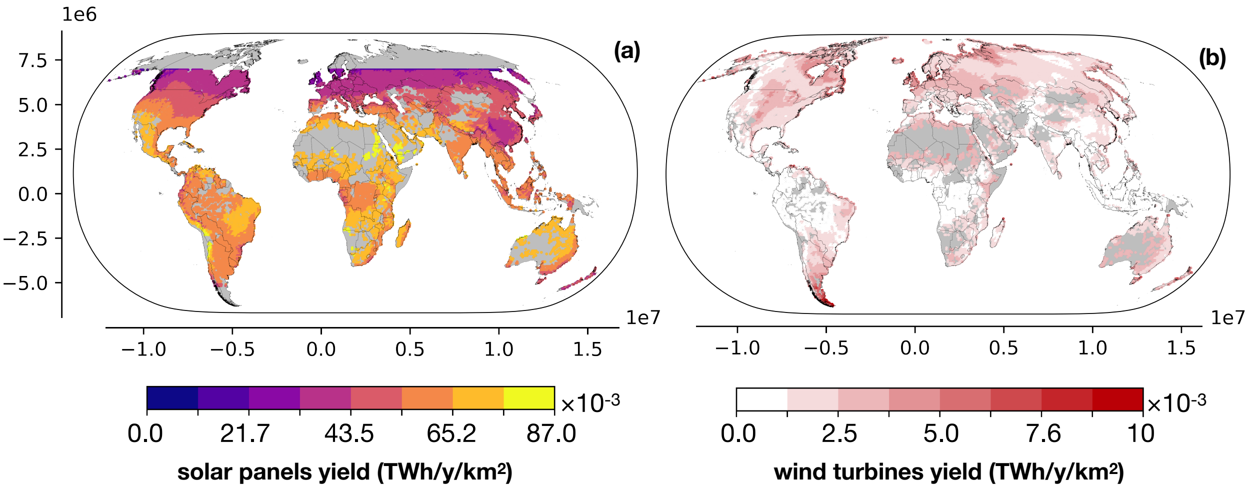

Figure S.2: Power production per unit of area from solar panels (a) and onshore wind turbines (b). The methodology is based on literature sources [32,33] and has been adjusted to the scope of this study by excluding cells within natural reserves [34] and areas with water scarcity [35]. For solar production, data limitations beyond +60° N and -45° S are inherited from the original dataset of solar irradiation [36]. The detailed data can be found in the **Supplementary Dataset**. The maps are created with the Matplotlib and Geopandas packages for Python [52,53].

Table S.3.a provides the power production yield for specific countries, specifically for solar panels and onshore wind turbines. These values are computed within this study using **Equations 1 and 2**, which are based on the methodology described in literature [32,33]. Complementary information can be found in **Table S.3.b** and **Table S.3.c** which provide the coefficients utilized in the computation of the power production yield, following the methodology outlined in literature [32,33].

Table S.3.a: Power production yield in sample countries. The power production from renewable technologies depends on the location of installation of the technologies. The values correspond to the area-weighted average of the power production over the grid cells within a country (**Figure S.3**).

**country - *c***	**solar panels - S_i^{solar} (TWh/km^2/y)**	**wind onshore - S_i^{wind} (TWh/km^2/y)**
Trinidad and Tobago	0.068	0.0034
Indonesia	0.061	0.0009
Canada	0.043	0.0039

Table S.3.b: Coefficients and resulting power production yield in sample countries. The table includes the coefficients used in the computation of power production yield at a cell level according to **Equations 1 and 2**, along with the corresponding power production yields in the sample countries. The methodology and coefficients are based on the approach described in literature [32,33].

variable	value	description
η^{solar} (-)	0.17	conversion efficiency solar panels
γ (-)	0.2	ground cover ratio of photovoltaic cells
I_i (MW/km^2)	Trinidad and Tobago: 221.96 - 235.54<br>Indonesia: 136.63 - 251.08<br>Canada: 59.54 - 167.08	solar irradiation
η_i^{wind} (-)	Trinidad and Tobago: 0.784 - 0.785<br>Indonesia: 0.618 - 0.877<br>Canada: 0.568 - 0.827	array efficiency of the wind park
c_i (-)	Trinidad and Tobago: 0.174 - 0.176<br>Indonesia: 0.0006 - 0.289<br>Canada: 0.026 - 0.409	capacity factor of onshore wind turbines
P_i (MW/km^2)	Trinidad and Tobago: 2.228 - 2.235<br>Indonesia: 0.42 - 2.84<br>Canada: 1.41 - 3.08	rated power per unit of area

Table S.3.c: Hydrogen production. Conversion of electricity into hydrogen is based on an electrolyzer conversion efficiency.

variable	value	description
$\eta^{electrolyzer}$ (-) [37]	0.62	electrolyzer efficiency

Table S.3.d: Coefficients for the quantification of eligible land for installation of solar panels and wind turbines. Coefficients depend on the typology of land cover.

eligibility coefficient f	forest [38]	agriculture [32,38]	urban [39]
**solar farms**	0	0.1	0.25
**wind farms**	0.1	0.7	0

Additional energy production could come from offshore wind turbines. However, within the scope of analyzing the impact of hydrogen production in terms of land and inland water demand, in this study offshore wind production was not considered.

S.4 Water demand

S.4.1 Water requirements

The total water volume required for electricity production from solar panels and wind turbines is estimated at 14 billion m^3 and 3 billion m^3 per year in 2020, and 59 billion m^3 and 14 billion m^3 per year in 2050. In comparison, the total volumes of water required for agriculture, industry and municipalities are 2853 billion m^3, 645 billion m^3 and 479 billion m^3 respectively [40], summing up to 3977 billion m^3. The water demand for hydrogen production varies between 1.5% and 0.4% of the total global water withdrawals, aligning with other studies that assume a 1.8% water withdrawal for meeting global hydrogen demand [41]. In the case of the United States, our findings consider a scenario of hydrogen demand of 74 Mt/y in 2050. Compared to other studies [42] that examine a wide range of hydrogen demand in a net-zero system (2.5 – 150 Mt/y), our findings represent a central case. Specifically, we estimate a water of approximately 1.7 billion m^3, which is approximately 13% of the freshwater consumption of the entire US energy system in 2014 [42].

Regarding water scarcity, hydrogen production from wind power is less water intensive compared to solar power, making it a preferable option for countries facing water scarcity. Conversely, hydrogen production from solar power is less land intensive compared to wind power, making it a preferable option for countries facing land scarcity. It is worth noting that this study does not account for the additional exacerbation of water scarcity caused by increased water demand for agriculture, industry, and municipal uses, which is expected to rise by one-third by 2050 [43,44].

For our estimates, we assume [45] an annual water demand for agriculture of 2853 billion m^3, industrial use of 645 billion m^3, and municipal use of 479 billion m^3 which are in line with values reported in the literature of 2769 billion m^3, 768 billion m^3 and 464 billion m^3 respectively [46]. The water requirements for producing of 1 kg of hydrogen is calculated based on the coefficients provided in **Table S.4**.

Table S.4: Coefficients used for the computation of the water requirements. Coefficients are based on an ecological footprint analysis for each technology. For solar panels, in the case of silicon-based photovoltaics, water requirements are mainly associated with the manufacturing of silicon (61%), the cell manufacturing (13%), the module assembly (11%), the construction of the production plant (7%) and the manufacturing of the inverter (6%) [47].

coefficient	value ($kg_{H2O}/kWh_{electricity}$)
LCA - solar panels - w^{solar} [47]	2.3 (124 l_{H2O}/kg_{H2})
LCA - onshore wind turbines - w^{wind} [47]	0.2 (11 l_{H2O}/kg_{H2})
LCA - water electrolysis - $w^{electrolyser}$ [48]	0.45 (24 l_{H2O}/kg_{H2})

S.4.2 Impact of water requirements for hydrogen production

Figure S.3 illustrates the additional water requirement in countries that are not affected by water scarcity, compared to the scenario without hydrogen production (see **Methods - Section 5.4 Water scarcity - Equations 9, 10**). Countries such as Russia, European nations, and Southern African countries like Namibia and Angola exhibit considerable increases in water demand. In these countries, the water requirement for hydrogen production represents more than 4% of the total water withdrawals for agriculture, industry, and municipal purposes. **Figure S.3** complements **Figure 5** in **Main text - Results** which illustrates the impact of water for hydrogen production in countries where water scarcity arises.

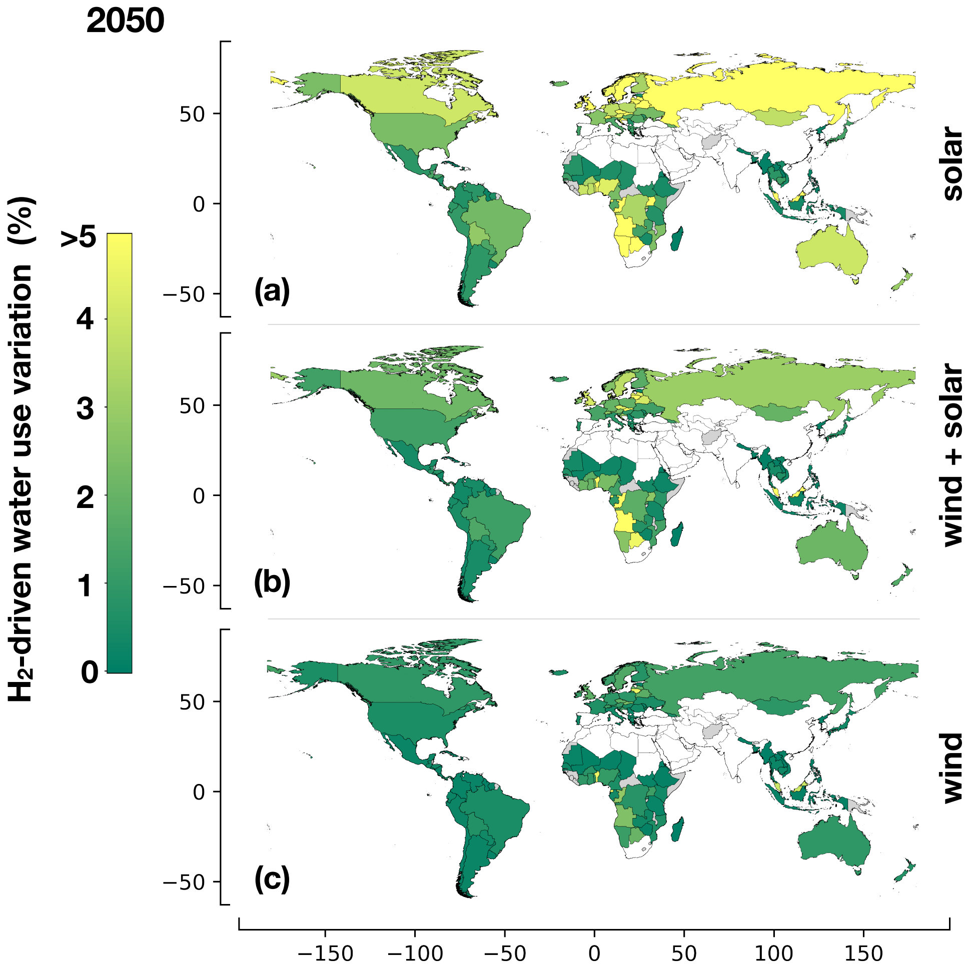

Figure S.3: Relative increase of water use due to additional water requirements for hydrogen production in 2050 through water electrolysis, compared to current water withdrawals for agricultural, industrial and municipal activities. Power production from (a) solar, (b) hybrid wind and solar and (c) onshore wind. Countries depicted without color indicate areas that experience water scarcity regardless of hydrogen demand. **Figure 5** illustrates the impact of water demand for hydrogen production in countries where water scarcity is present. It is worth noting that the demand for water in hydrogen production alone does not lead to water scarcity where it is not already present. The gray-colored countries indicate areas for which data is unavailable. The maps are created with the Matplotlib and Geopandas packages for Python [52,53].

S.5 Combination of solar and wind

The energy production per unit area from hybrid solar photovoltaics and wind turbine systems is computed as:

$$S_i^{wind+solar} = 0.6\, S_i^{wind} + 0.4\, S_i^{solar} \qquad \forall\, i \in N \tag{11}$$

with:

- $S_i^{wind+solar}$ (TWh/km^2/y) yearly energy production from hybrid wind and solar systems in cell *i*.

Here, we choose hybrid systems with a production composed of 60% wind turbines and 40% solar panels, corresponding to the minimization of the storage capacity needed [49]. In the following, we show the list of figures presented in the article for the case of energy production per unit area from hybrid solar photovoltaics and wind turbine systems.

In the following, we present the corresponding figures for the case of hybrid solar and wind systems, which are equivalent to those presented in the main text.

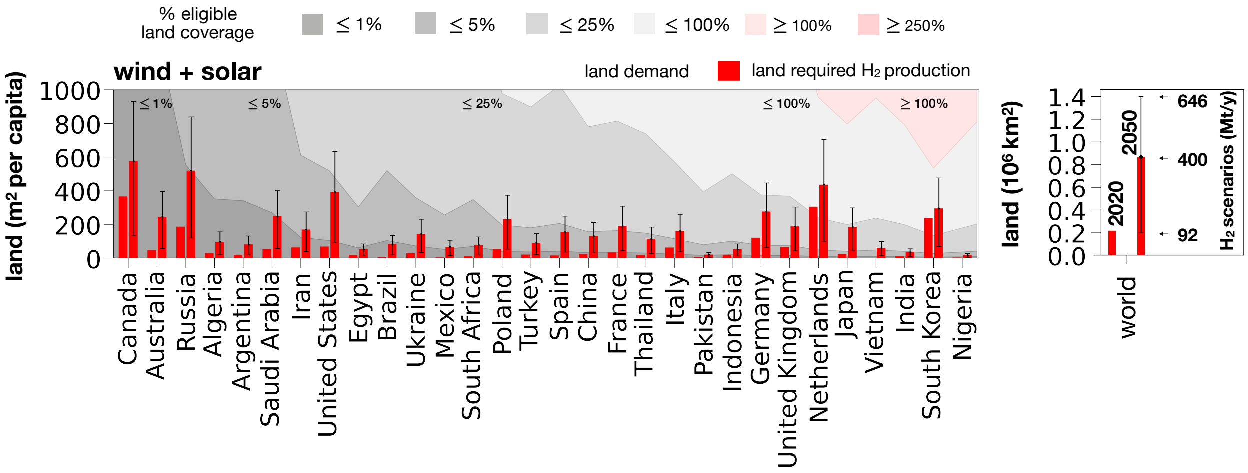

Figure S.4: Per capita land requirements to meet the hydrogen demand in 2020 and 2050 based on hybrid solar and wind systems. The interval bars represent the range of land required in 2050 based on different reference and extreme scenarios of global hydrogen demand in 2050: 92 Mt/y (smallest), 400 Mt/y (reference), 646 Mt/t (largest). The gray areas (and pink areas) indicate fractions (and multiples) of the eligible land, defined in **Methods** - **Section 5.4 Land scarcity** as the 100% threshold, for comparison with the land requirements for hydrogen demand in each country. Different energy generation technologies require varying amounts of land for the same amount of hydrogen demand. Countries are selected based on the highest total demand of hydrogen in 2050 and are ordered according to the amount of eligible land for solar panels (excluding Trinidad and Tobago for scaling reasons).

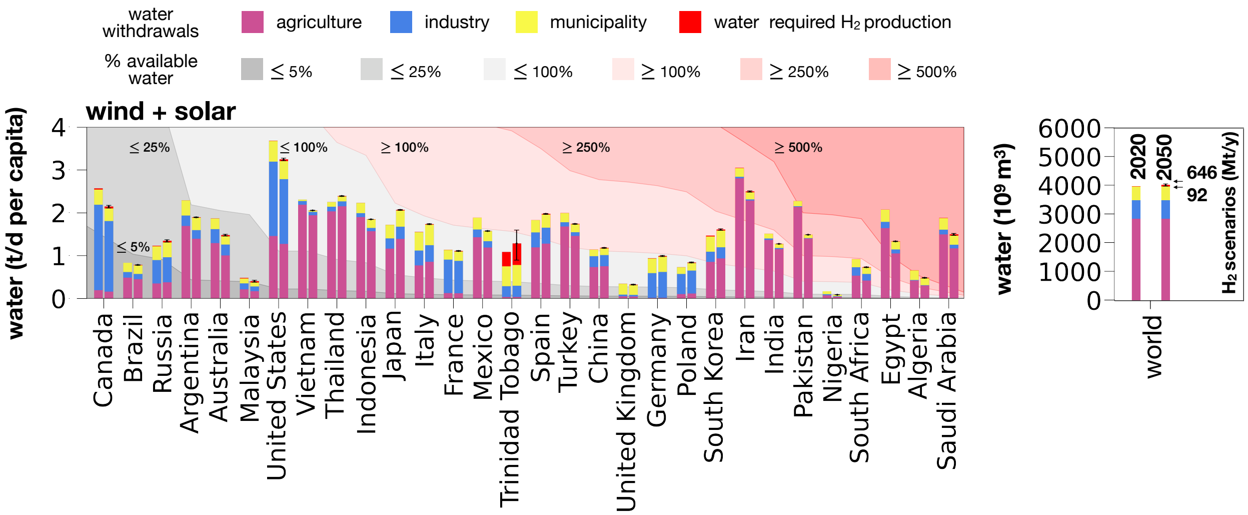

Figure S.5: Water withdrawals per capita to satisfy hydrogen demand in 2020 and 2050, based on hybrid solar and wind systems. The gray areas represent the fractions of available water resources (water resources minus environmental flow requirements) in each country, corresponding to the water withdrawals. The red areas indicate water withdrawals exceeding the water availability limit (100%, 250%, and 500%). The purple, blue and yellow stacks represent water withdrawal in agriculture, industry and municipality, respectively. The red stack represents the water required for hydrogen demand in 2020 and 2050. Total water withdrawals exceeding 100% indicate the presence of water scarcity. The countries were selected from the top 30 in terms of total hydrogen demand in 2050 and are ordered according based on the amount of available water.

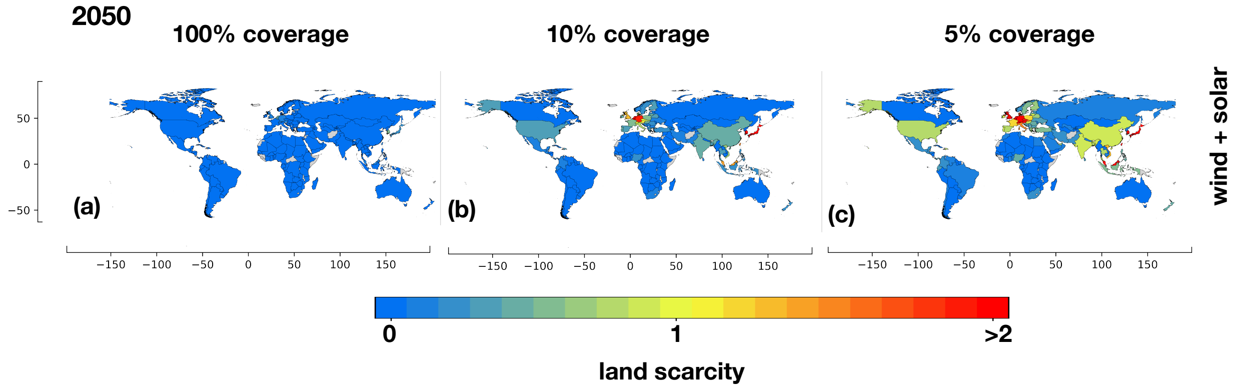

Figure S.6: Land scarcity induced by hydrogen production in 2050 across worldwide countries, considering various fractions of land coverage (a, b, c) for hybrid solar and wind systems. The degree of land scarcity is closely tied to the assumed eligible land coverage ($f^{coverage}$) for renewable technologies in each country, reflecting economic and socio-political constraints. Countries depicted in gray lacked available data. The maps are created with the Matplotlib and Geopandas packages for Python [52,53].

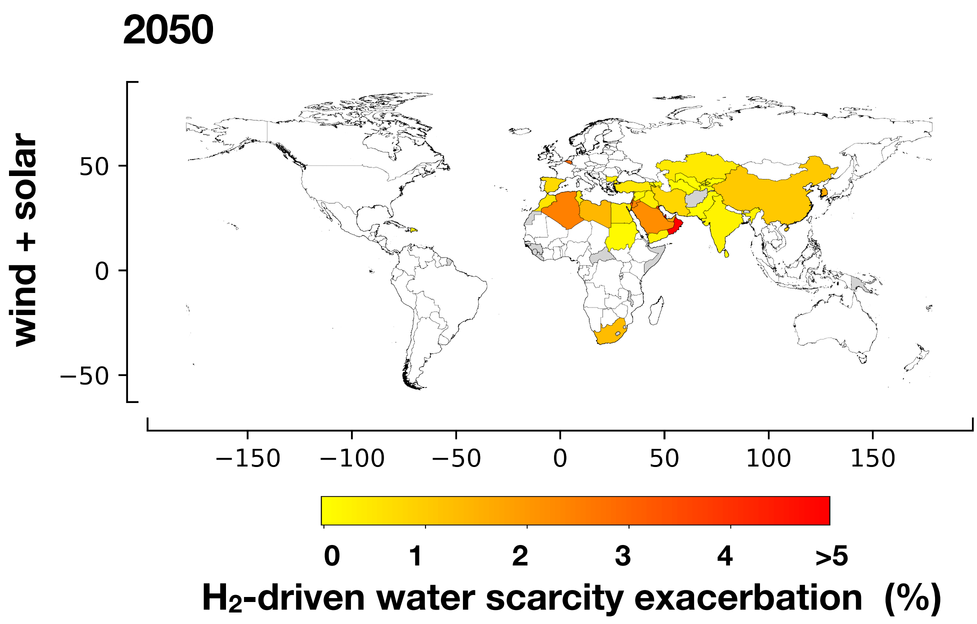

Figure S.7: Exacerbation of water scarcity resulting from additional water requirements for hydrogen production in 2050 through water electrolysis, compared to current water withdrawals for agricultural, industrial and municipal activities. The demand for water in hydrogen production alone does not create water scarcity in countries where it is not already present. However, the additional water demand for hydrogen production can exacerbate scarcity in countries already affected by water scarcity. Countries without color in the figure do not experience water scarcity. Gray-colored countries indicate unavailable data. The maps are created with the Matplotlib and Geopandas packages for Python [52,53].

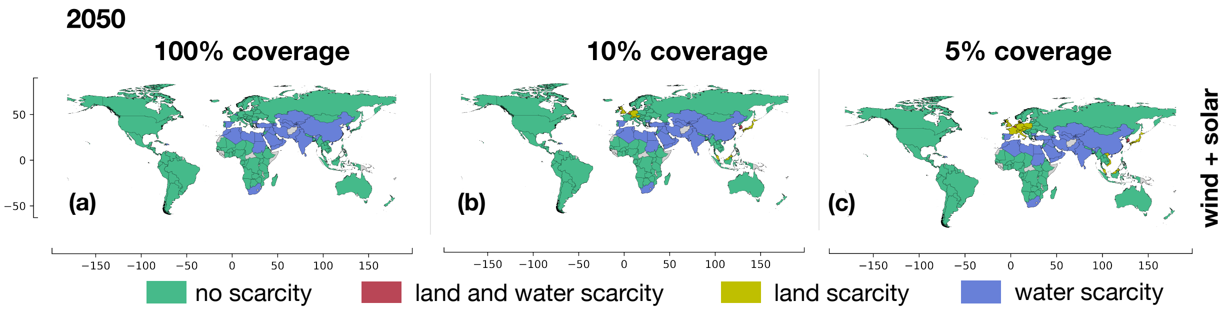

Figure S.8: Land and water scarcity induced by projected hydrogen production in 2050 for all countries worldwide, considering various fractions of land coverage (a, b, c) for hybrid solar and wind systems. The color-coded regions represent the scarcity status: green for no scarcity, red for both land and water scarcity, yellow for land scarcity only, and blue for water scarcity only. Gray-colored countries indicate unavailable data. The maps are created with the Matplotlib and Geopandas packages for Python [52,53].

S.6 Material requirements

Requirements of material can be derived from the LCA of solar panels [47], onshore wind turbines [47] and Proton Exchange Membrane electrolysers [50], here reported from literature sources.

Table S.5: Material requirements.

mineral	solar panels (g/MWh)	wind turbines (g/MWh)	electrolyser (g/MW)
Silicon - Zinc	67	9	0
Nickel	24	67	0
Manganese	60	55	0
Copper	170	24	7
Cobalt	0	70	0
Aluminum	292	52	41
Titanium	0	0	283
Steel	0	0	70

S.7 Land use - countries detail

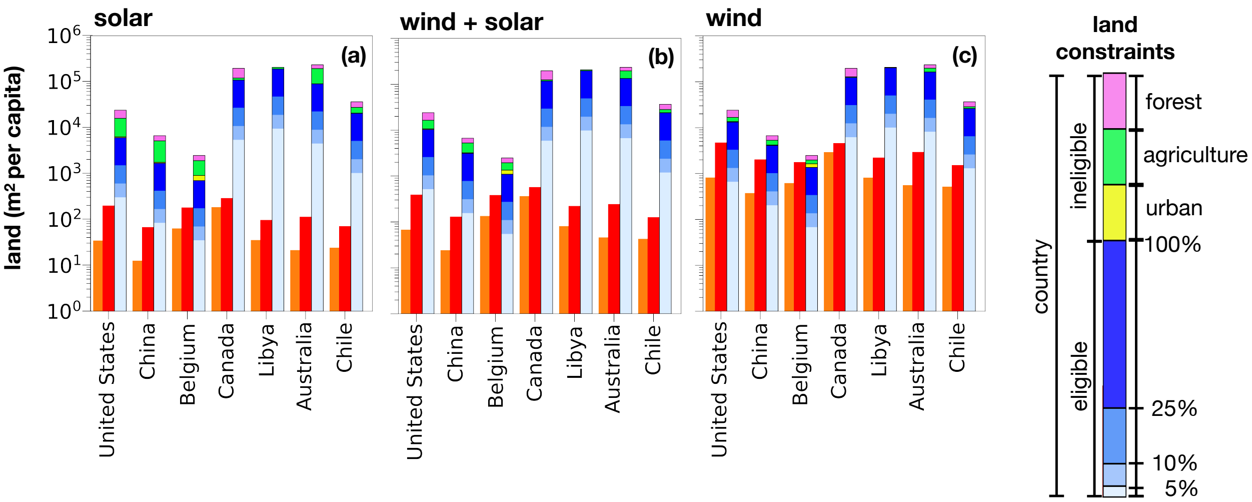

Figure S.9: Land demand per sector per country for a limited number of countries. Power production from (a) solar, (b) hybrid wind and solar and (c) onshore wind. Orange and red columns represent the land required for hydrogen production in 2020 and 2050 and are compared with the amount of land which can be covered with renewable technologies for electrolytic hydrogen production (eligible).

S.8 Water requirements - countries detail

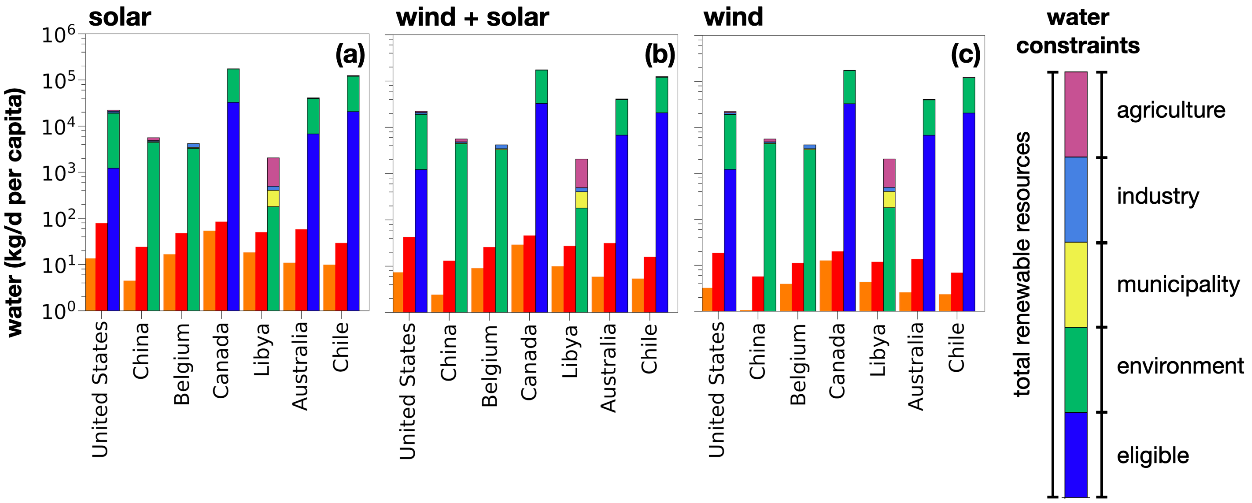

Figure S.10: Water withdrawals per sector per country for a limited number of countries. Power production from (a) solar, (b) hybrid wind and solar and (c) onshore wind. Orange and red columns represent the water required for hydrogen production in 2020 and 2050 and are compared with the amount of water that can be sustainably used (eligible).

S.9 Sensitivity number of countries with land and water scarcity

The extent of land and water scarcity affecting countries for power production from solar panels, hybrid solar and wind systems, and wind turbines depends on the assumed parameter of eligible land coverage ($f^{coverage}$). **Figure S.11** illustrates the varying number of countries affected across a wide range of $f^{coverage}$ values, ranging from 1% to 95%. The number of countries with concurrent presence of land and water scarcity is 14 for solar panel power production and 28 for wind turbines, when assuming only 1% of eligible land coverage. As the value of coverage increases, the concurrent presence of land and water scarcity decreases substantially. Hybrid wind and solar systems present an intermediate case in terms of both water and land use. Due to the lower water requirements of power production from wind turbines, the number of countries affected by both land and water scarcity slightly decreases compared to the case of solar panels.

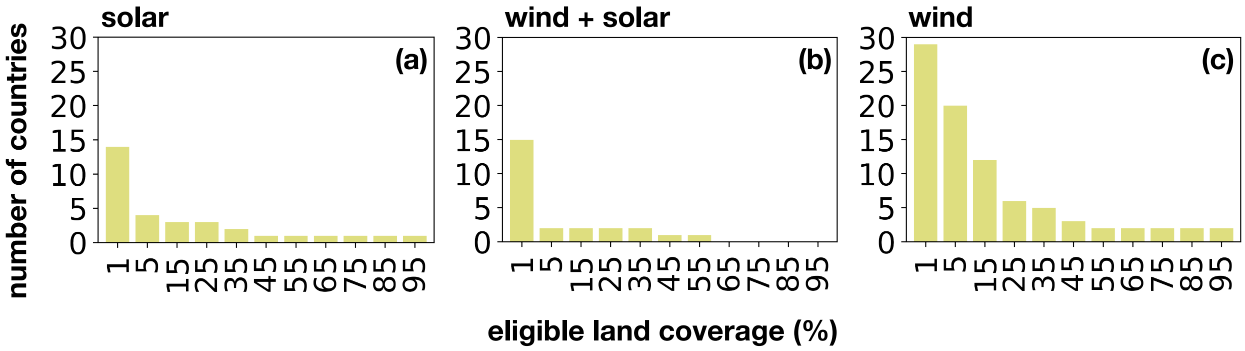

Figure S.11: Number of countries affected by concurrent land and water scarcity for power production from (a) solar panels, (b) hybrid solar and wind systems, and (c) wind turbines. The figure illustrates the

sensitivity of the number of countries experiencing both land and water scarcity based on the eligible land coverage parameter.

The predictions for hydrogen demand in 2050 vary considerably across scenarios, ranging from 3% to 12% of the global total final energy consumption, corresponding to a range of 92 Mt/y to 646 Mt/y [51]. In this analysis, we examine the implications of this variation in hydrogen demand on global land and water scarcity. Based on the relationship between the number of countries affected by the concurrent land and water scarcity highlighted in **Figure S.11**, we compare three cases of hydrogen demand, assuming a coefficient of eligible land coverage equal to 10%. Under the scenario with the smallest demand of 92 Mt/y in **Figure S.12 (a, b, c)**, the number of countries experiencing both land and water scarcity is 0 for power production from solar panels, 1 (South Korea) for hybrid wind and solar systems and 3 (Belgium, India and South Korea) for wind turbines. Moving to the reference demand scenario of 400 Mt/y in **Figure S.12 (d, e, f)** this number increases to 3 (Belgium, South Korea and Trinidad and Tobago) for solar panels, 2 (Belgium and South Korea) for hybrid wind and solar systems and 14 (including China, United Arab Emirates, Iran) for wind turbines. Finally, under the largest demand scenario of 646 Mt/y in **Figure S.12 (g, h, i)**, this number further increases to 3 (Belgium, South Korea and Trinidad and Tobago) for solar panels, 3 for hybrid wind and solar systems and 19 (including China, Oman, South Africa) for wind turbines.

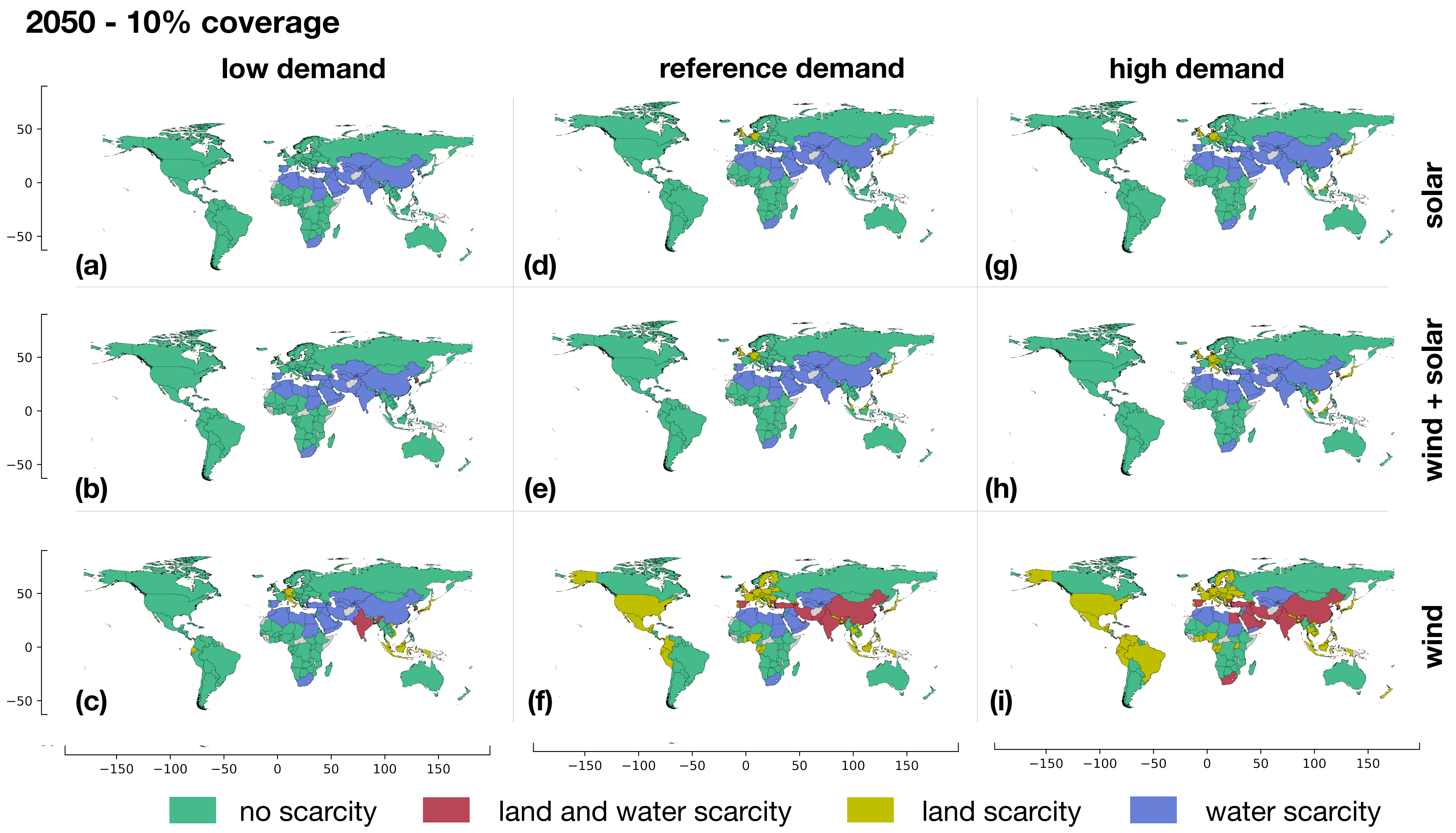

Figure S.12: Land and water scarcity resulting from projected hydrogen demand in 2050 for all countries worldwide. Three demand scenarios are considered: (a, b, c) low - 92 Mt/y, (d, e, f) reference - 400 Mt/y, and (g, h, i) high - 646 Mt/t. The color-coded regions depict the scarcity status: green for no scarcity, red for both land and water scarcity, yellow for land scarcity only, and blue for water scarcity only. Gray-colored countries indicate areas with unavailable data. Power production from (a,d,g) solar panels, (b,e,h) hybrid solar and wind from a combination of 60% solar panels and 40% wind turbines, and (c,f,i) wind turbines. The maps are created with the Matplotlib and Geopandas packages for Python [52,53].

S.10 Potential hydrogen exporters and importers

We define the net potential of a country to become an exporter or importer of electrolytic hydrogen using the following equation:

$$P_c = f^{coverage} A_c S_c - E_c - D_c / \eta^{electrolyzer} \qquad \forall\, c \in C \qquad (11)$$

Here, D_c represents the total demand of hydrogen in country c (see **Methods – Section 5.1 Hydrogen demand**); E_c is the electricity demand for direct consumption in country c (see **Methods – Section 5.2 Electricity demand**); $f^{coverage}$ is the fraction of eligible land coverage; A_c is the land area eligible for solar panels installation in country c, A_c^{solar}, or onshore wind parks installation, A_c^{wind}, required to run water electrolysis in country c. S_c denotes the amount of energy generated per unit of area from solar panels (S_c^{solar}) or wind turbines (S_c^{wind}) and $\eta^{electrolyser}$ is the conversion efficiency of a water electrolyzer in converting electricity to hydrogen. The country-specific values of energy production (S_c^{solar}, S_c^{wind}) were computed by considering the grid cells with the top 25% energy production within the boundary of country c, and then averaging the values over the cell area (see **Methods – Section 5.3 Energy production**). While $f^{coverage}$ is a country-specific parameter, we assume a reference value of 10% for all the countries. Under this constraint, and based on the country-specific power production potential from solar panels and wind turbines, the demand of hydrogen and the demand of electricity per country, we identify the countries with a potential surplus of power for hydrogen export and those with a potential deficit, requiring hydrogen import.

Figure S.13 highlights the countries with the greatest potential for hydrogen export or need of hydrogen import. In the case of power production from solar panels, Russia, Australia, Canada, countries in North and South Africa, the United States and South American countries present the largest export potential (**Figure S.13 (c)**). Countries with the largest deficit in case of power production from solar panels include China, European countries, Japan, South Korea, and Trinidad and Tobago (**Figure S.13 (a)**). When it comes to power production from wind turbines, the countries with the highest potential for export include Canada, Australia, countries in North and South Africa, and South American countries (**Figure S.13 (d)**). Conversely, countries with the largest deficit in case of wind power production include China, the United States, Japan, South Korea, European countries and South American countries (**Figure S.13 (b)**)

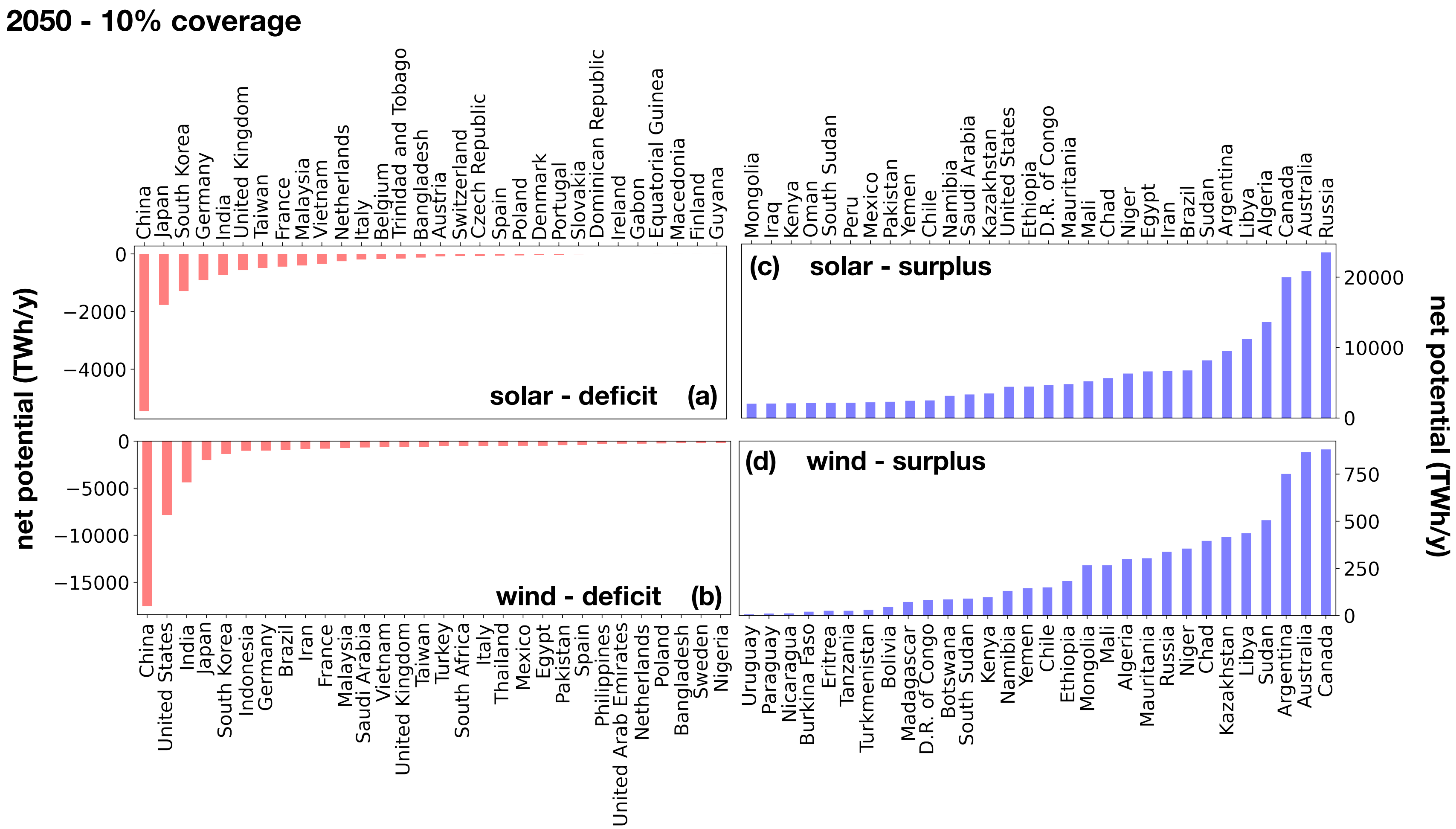

Figure S.13: Surplus and deficit of renewable power potential for electrolytic hydrogen production. Net potential is computed as the difference of renewable power potential minus electricity demand for direct consumption and electrolytic hydrogen production in 2050. The countries shown are limited to (c, d) the top 20 countries for surplus in net potential and (a, b) the 20 countries with the largest deficit in net potential.

SYMBOLS

Table S.6: Summary of symbols, Greek letters and abbreviations from the main text.

**symbols**	
$f^{forest}, f^{agriculture}, f^{urban}, f^{coverage}$	eligibility coefficients per typology of land and fraction of eligible land coverage
g	variation of population
e	fraction of global emissions
s	fraction of total final consumption
r	regional share of production of ammonia
t	regional share of production of methanol
S, S^{solar}, S^{wind}	amount of energy generated per unit area from solar panels or wind turbines
I	yearly average solar irradiation
C	capacity factor wind turbines
P	rated power per square meter wind turbines
$A, A^{solar}, A^{wind}, A^{forest}, A^{agriculture}, A^{urban}$	area covered by solar panels, onshore wind turbines, forests, agriculture or buildings
$A^{eligible}, A^{coverage}$	area eligible or practically covered
D	total demand of hydrogen
$w^{solar}, w^{wind}, w^{electrolyzer}$	water per unit of electricity generated or consumed
W	total water requirements
LS	land scarcity
WA	water availability
WR	total renewable water resources
EFR	environmental flow requirements
WW, $WW^{hydrogen}$	water withdrawals for power production and hydrogen production
WS	water scarcity
**Greek letters**	
$\eta^{solar}, \eta^{wind}, \eta^{electrolyzer}$	efficiency of solar panels, wind turbines, electrolyzers
γ	ground cover ratio solar panels
**abbreviations**	
GHG	greenhouse gas
HVCs	high value chemicals
NG	natural gas
TFC	total final energy consumption
MENA	North Africa, Middle East

SUPPLEMENTARY REFERENCES

1. International Energy Agency. Net Zero by 2050. https://www.iea.org/reports/net-zero-by-2050 (2021).
2. Gabrielli, P., Poluzzi, A., Kramer, G.J., Spiers, C., Mazzotti, M. & Gazzani, M. Seasonal energy storage for zero-emissions multi-energy systems via underground hydrogen storage. *Renewable and Sustainable Energy Reviews* **121**, 109629 (2020).
3. Chiesa, P., Lozza, G. & Mazzocchi, L. Using hydrogen as gas turbine fuel. *J. Eng. Gas Turbines Power* **127(1)**, 73-80 (2005).
4. Byers, L., Friedrich, J., Hennig, R., Kressig, A., Li, X., McCormick, C. & Valeri, L.M. A global database of power plants. *World Resources Institute* **18**. https://www.wri.org/research/global-database-power-plants (2018).
5. International Energy Agency. World Energy Balances: Overview. https://www.iea.org/reports/world-energy-balances-overview (2021).
6. Global Infrastructure Emission Database, GID Database, http://gidmodel.org.cn/.
7. Tong, D., Zhang, Q., Zheng, Y., Caldeira, K., Shearer, C., Hong, C., Qin, Y. & Davis, S.J. Committed emissions from existing energy infrastructure jeopardize 1.5 C climate target. *Nature* **572(7769)**, 373-377 (2019).
8. Liu, J., D. Tong, Y. Zheng, J. Cheng, X. Qin, Q. Shi, L. Yan, Y. Lei & Q. Zhang, Carbon and air pollutant emissions from China's cement industry 1990 - 2015: trends, evolution of technologies and drivers, *Atmos. Chem. Phys.* **21**, 1627–1647 (2021).
9. International Energy Agency. The Future of Petrochemicals. https://www.iea.org/reports/the-future-of-petrochemicals (2018).
10. Parkinson, B., Balcombe, P., Speirs, J.F., Hawkes, A.D. & Hellgardt, K. Levelized cost of CO_2 mitigation from hydrogen production routes. *Energy & Environmental Science* **12(1)**, 19-40 (2019).
11. Pflugmann, F. & Blasio, N.D. Geopolitical and Market Implications of Renewable Hydrogen. New Dependencies in a Low-Carbon Energy World. https://www.belfercenter.org/publication/geopolitical-and-market-implications-renewable-hydrogen-new-dependencies-low-carbon (2020).
12. Bauer, C., Treyer, K., Antonini, C., Bergerson, J., Gazzani, M., Gencer, E., Gibbins, J., Mazzotti, M., McCoy, S.T., McKenna, R. & Pietzcker, R. On the climate impacts of blue hydrogen production. *Sustainable Energy & Fuels* **6(1)**, 66-75 (2022).
13. Yu, J., Hmiel, B., Lyon, D.R., Warren, J., Cusworth, D.H., Duren, R.M., Chen, Y., Murphy, E.C. & Brandt, A.R. Methane Emissions from Natural Gas Gathering Pipelines in the Permian Basin. *Environmental Science & Technology Letters* **9(11)**, 969-974 (2022).
14. Miller-Wang, A. China's hydrogen development: A tale of three cities. *OIES Paper* **5** (2023).
15. Lester, T. The plan to deliver net zero: The Australian way. *Appita Magazine* **4**, 38-39 (2021).
16. COAG Energy Council Hydrogen Working Group. Australia's national hydrogen strategy. (2019).
17. Ministre de l'Économie, des Finances et de la Souveraineté industrielle et numérique. Dossier de Presse - Stratégie nationale pour le développement de l'hydrogène décarboné en France. https://www.entreprises.gouv.fr/fr/strategies-d-acceleration/strategie-nationale-pour-developpement-de-l-hydrogene-decarbone-france (September 2020).
18. Ministero dell'Ambiente e della Sicurezza Energetica, Strategia Nazionale Idrogeno Linee Guida Preliminari. https://www.mase.gov.it/pagina/strategia-nazionale-idrogeno-sni (2020).
19. Hoja de Ruta del Hidrógeno. Ministerio para la Transición Ecológica y el Reto Demográfico. https://www.miteco.gob.es/es/ministerio/planes-estrategias/hidrogeno/default.aspx.
20. Federal Ministry for Economic Affairs and Energy Public Relations Division. The National Hydrogen Strategy. https://www.bmwk.de/Redaktion/EN/Publikationen/Energie/the-national-hydrogen-strategy.html (2020).
21. Communication from the Commission to the European Parliament, the European Council, the Council, the European Economic and Social Committee and the Committee of the regions. REPowerEU Plan. https://eur-lex.europa.eu/legal-content/EN/TXT/?uri=COM%3A2022%3A230%3AFIN&qid=1653033742483 (2022).
22. Belgian Federal Hydrogen Strategy. Vision and strategy. https://economie.fgov.be/en/themes/energy/belgian-federal-hydrogen (2022).
23. Chance, C. Focus on hydrogen: Japan's energy strategy for hydrogen and ammonia. *Clifford Chance* (2022).
24. Japan Ministry of Economy, Trade and Industry. Basic Hydrogen Strategy, Key Points. https://prod5.assets-cdn.io/event/6605/assets/8377898940-76681eb222.pdf (2020).

25. Infrastructure Investment and Jobs Act. H.R.3684 - 117th Congress (2021-2022). 117th Cong. https://www.congress.gov/bill/117th-congress/house-bill/3684/text.
26. U.S. Department of Energy, DOE National Clean Hydrogen Strategy and Roadmap, Draft, September 2022, https://www.hydrogen.energy.gov/pdfs/clean-hydrogen-strategy-roadmap.pdf.
27. House of Representatives, Congress. H.R. 812 (IH) - Inflation Reduction Act of 2023.
28. Energy Information Administration (EIA). https://www.eia.gov/international/data/world (2021).
29. United Nations, Department of Economic and Social Affairs, Population Division. World Population Prospects 2022, Online Edition, https://population.un.org/wpp/Download/Standard/Population/ (2022).
30. Enerdata. Global Energy & Climate Outlook 2050. https://eneroutlook.enerdata.net/forecast-world-electricity-consumption.html (2022).
31. U.S. Energy Information Administration (EIA). https://www.eia.gov/international/data/world (2021).
32. Dupont, E., Koppelaar, R. & Jeanmart, H. Global available solar energy under physical and energy return on investment constraints. *Applied Energy* **257**, 113968 (2020).
33. Dupont, E., Koppelaar, R. and Jeanmart, H. Global available wind energy with physical and energy return on investment constraints. *Applied Energy* **209**, 322-338 (2018).
34. UNEP-WCMC and IUCN. Protected Planet: The World Database on Protected Areas (WDPA) and World Database on Other Effective Area-based Conservation Measures (WD-OECM), https://www.protectedplanet.net/en (2022).
35. Hofste, R.W., Kuzma, S., Walker, S., Sutanudjaja, E.H., Bierkens, M.F.P., Kuijper, M.J.M., Sanchez, M.F., Van Beek, R., Wada, Y., Galvis Rodríguez, S. & Reig, P. Aqueduct 3.0: Updated Decision- Relevant Global Water Risk Indicators. Technical Note, World Resources Institute. https://www.wri.org/publication/aqueduct-30
36. ESMAP. Global Solar Atlas 2.0. Technical Report, World Bank. https://globalsolaratlas.info/map (2019).
37. Everall, J., & Ueckerdt, F. Electrolyser CAPEX and efficiency data for: Potential and risks of hydrogen-based e-fuels in climate change mitigation. doi:10.5281/zenodo.4619892 (2021).
38. Hoogwijk, M.M. On the global and regional potential of renewable energy sources. Doctoral dissertation (2004).
39. Melius, J., Margolis, R. & Ong, S. Estimating rooftop suitability for PV: a review of methods, patents, and validation techniques (2013).
40. FAO. AQUASTAT Database. https://www.fao.org/aquastat/en/ (2022).
41. Newborough, M. & Cooley, G. Green hydrogen: water use implications and opportunities. *Fuel Cells Bulletin* **2021(12)**, 12-15 (2021).
42. Grubert, E. Water consumption from electrolytic hydrogen in a carbon-neutral US energy system. *Cleaner Production Letters* **4**, 100037 (2023).
43. United Nations World Water Assessment Program (WWAP) UN-Water. The United Nations World Water Development Report 2018: Nature-Based Solutions for Water. https://unesdoc.unesco.org/ark:/48223/pf0000261424 (2018).
44. Boretti, A. & Rosa, L. Reassessing the projections of the world water development report. *NPJ Clean Water* **2(1)**, 1-6.
45. FAO. AQUASTAT Database. https://www.fao.org/aquastat/en/ (2022).
46. International Renewable Energy Agency. Geopolitics of the Energy Transformation: The Hydrogen Factor. https://www.irena.org/publications/2022/Jan/Geopolitics-of-the-Energy-Transformation-Hydrogen (2022).
47. UNECE. Carbon Neutrality in the UNECE Region: Integrated Life-cycle Assessment of Electricity Sources, https://unece.org/sites/default/files/2022-04/LCA_3_FINAL%20March%202022.pdf (2022).
48. Lampert, D.J., Cai, H., Wang, Z., Keisman, J., Wu, M., Han, J., Dunn, J., Sullivan, J.L., Elgowainy, A. & Wang, M. Development of a life cycle inventory of water consumption associated with the production of transportation fuels. *Argonne National Lab*, No. ANL/ESD-15/27 (2015).
49. Heide, D., Von Bremen, L., Greiner, M., Hoffmann, C., Speckmann, M. & Bofinger, S. Seasonal optimal mix of wind and solar power in a future, highly renewable Europe. *Renewable Energy* **35(11)**, 2483-2489 (2010).
50. Bareiß, K., de la Rua, C., Möckl, M. & Hamacher, T. Life cycle assessment of hydrogen from proton exchange membrane water electrolysis in future energy systems. *Applied Energy* **237**, 862-872 (2019).
51. Riemer M., Zheng L., Eckstein J., Wietschel M., Pieton N. & Kunze R. Future hydrogen demand: A cross-sectoral, global meta-analysis. *HYPAT Working Paper* **04/2022**, https://www.isi.fraunhofer.de/content/dam/isi/dokumente/cce/2022/HYPAT_Working_Paper_04_2022_Future_hydrogen_demand.pdf (2022).

52. J. D. Hunter, Matplotlib: A 2D Graphics Environment. Computing in Science & Engineering **9(3)**, 90-95. https://matplotlib.org/stable/index.html (2007).
53. K. Jordahl, J. Van den Bossche, M. Fleischmann, J. Wasserman, J. McBride, J. Gerard, J. Tratner, M. Perry, A. G. Badaracco, C. Farmer, G. A. Hjelle, A. D. Snow, M. Cochran, S. Gillies, L. Culbertson, M. Bartos, N. Eubank, A. Bilogur, S. Rey, C. Ren, D. Arribas-Bel, L. Wasser, L. J. Wolf, M. Journois, J. Wilson, A. Greenhall, C. Holdgraf, F. Leblanc. geopandas/geopandas: v0.8.1. Zenodo. http://doi.org/10.5281/zenodo.3946761 (2020).
